# Challenges in Implementing Sendai Framework for Disaster Risk Reduction in Poland

**DOI:** 10.3390/ijerph16142574

**Published:** 2019-07-18

**Authors:** Krzysztof Goniewicz, Frederick M. Burkle

**Affiliations:** 1 Department of Security Studies, Polish Air Force Academy, Dywizjonu 303 Street, no 35, 08521 Dęblin, Poland; 2 Harvard Humanitarian Initiative, T.H. Chan School of Public Health, Harvard University, Cambridge, MA 02138, USA

**Keywords:** disaster risk reduction, disaster prevention and preparedness, Sendai framework, sustainable development

## Abstract

Currently, weather conditions and extreme weather are becoming more frequent and more intense. Along with climate change, the vulnerability of society and individual regions to the risk of various types of threats also increases. The objectives of “The Sendai Framework for Disaster Risk Reduction 2015–2030”, are the first global political frameworks of the United Nations program designed for the period post-2015. The original priority objectives of the Framework are: Understanding disaster risk, strengthening disaster risk governance to manage disaster risk, investing in disaster risk reduction for resilience, and enhancing disaster preparedness for effective response, and to “Build Back Better” in recovery, rehabilitation, and reconstruction. The provisions contained in the document are an essential step towards building global political coherence with an apparent reference to health, development, and climate change. The article is briefly reporting current Disaster Risk Reduction related programs and policies in Poland, contributions as part of The Sendai Framework for Disaster Risk Reduction implementation, and its challenges.

## 1. Introduction

The Sendai Framework for Disaster Risk Reduction 2015–2030 (SFDRR) is the first major agreement since the Yokohama Strategy (1994) and the Hyogo Framework for Action (2005–2015). The SFDRR development agenda, which sets it apart from the previous frameworks, includes four priorities for action and seven targets. The four priorities for focused action within and across sectors by States at local, national, regional and global levels are: (1) Understanding disaster risk, (2) strengthening disaster risk governance to manage disaster risk, (3) investing in disaster risk reduction for resilience, and (4) enhancing disaster preparedness for effective response and to “Build Back Better” in recovery, rehabilitation and reconstruction. The SFDRR 7 Global Targets (and their 38 indicators) include A: Substantially reduce global disaster mortality by 2030, aiming to lower the average per 100,000 global mortality between 2020–2030 compared with 2005–2015, B: Substantially reduce the number of affected people globally by 2030, aiming to lower the average global figure per 100,000 between 2020–2030 compared with 2005–2015, C: Reduce direct disaster economic loss in relation to global gross domestic product (GDP) by 2030, D: Substantially reduce disaster damage to critical infrastructure and disruption of basic services, among them health and educational facilities, including through developing their resilience by 2030, E: Substantially increase the number of countries with national and local disaster risk reduction strategies by 2020, F: Substantially enhance international cooperation to developing countries through adequate and sustainable support to complement their national actions for implementation of this framework by 2030, G: Substantially increase the availability of and access to multi-hazard early warning systems and disaster risk information and assessments to the people by 2030 [1].

Throughout the world, both the number of natural hazards, and direct and indirect mortality and morbidity increase every year. It is forecast that Poland will increasingly be exposed to the adverse effects of climate change and essential infrastructure losses [1,2,3]. Research supports that if root causes related to prevention and preparedness are not immediately addressed, there will be a predictable exponential rise in all health-related consequences even if the strength of the weather-related disasters remain the same. The most effective defense against this type of phenomena is a coherent disaster risk management strategy, taking into account the role of a central administration, local governments, and the private sector. In Poland, although awareness of potential threats is growing, the likelihood of natural hazards is underestimated. Currently, there is also no strategy to manage the reduction of risk of natural hazards [2,3].

A global agreement designated by Poland and other nations, named the Sendai Framework for Disaster Risk Reduction (SFDRR) 2015–2030, clearly indicates the need to achieve goals, including adoption of plans to reduce the risk of natural disasters at local level, activation of local communities’ activities, and cooperation of public administration with universities [4,5,6,7].

Multiple studies indicate the need to implement various strategies to reduce damage caused by natural phenomena, such as heavy rain, floods, frost, heat waves, droughts, forest fires, storms, and hailstorms. These strategies must be implemented at various levels of management including grassroots activities of local communities. Whereas central prevention and the investment in technical protective infrastructure and activities dominate in Poland today, there is a lack of communication with local communities and enterprises in the period preceding disasters. This includes the implementation of solutions by individuals and small businesses, as well as informal education conducted locally, and more extensive formal education in the scope of risk, and the consequences of natural disasters. The proven principles of spatial planning in areas threatened by dangerous natural phenomena should be re-verified [8,9,10].

Following the recommendations contained in the SFDRR, the development of disaster risk management strategies must be based on several assumptions. One is to cease the further increase in the exposure, i.e., the increase in the number of inhabitants and the value of assets exposed to natural disasters, especially in flood plains and landslides. It is said that “On the side of the State, there is a need to reduce ad-hoc ex-post actions and increase the pressure to develop long-term, cost-effective solutions in cooperation with the insurance industry” [4].

## 2. Cross-Sectoral Approaches

In Poland, more and more cross-sectoral activities are being undertaken for effective climate risk management. Public institutions currently lead in the implementation of these related activities. They are seen as legitimate and progressive to the mutual exchange of data, information, and knowledge, which must be seen and accepted as evidence of a common orientation to ensure the security of the state and its citizens regardless of the circumstances. Seasoned disaster managers stress civil planning implemented as part of the crisis management system, critical infrastructure protection standards, developing issues of business continuity management, as well as scientific and research activities [11,12].

Bearing in mind the so-called “Flood of the millennium in Poland” from 1997, which caused losses to the order of 3.34 billion U.S. dollars, additional floods from May 2010, including annual flooding in the spring and multiple smaller floods, violent storms, rainfall, and hailstorm are observed to be growing in activity of insurance companies and citizens’ awareness. An insufficient percentage of insured properties, in particular, those of individuals and local governments, has resulted in the central authorities taking on a significant burden of liquidation of damages during natural disasters. This requires an increase in insurance news and the level of education in the field of risk management. Such action is less effective than creating a system in which the share of the insurance sector is larger. However, this requires an increase in insurance news and the level of education in the field of risk management. As such, the following programs have been initiated.

Insurers belay more and more the risks associated with climate change, approaching the subject very comprehensively. At the same time, they gather more and more accurate data based on which risk can be more consciously and effectively managed [13]. This belies an increasing trend toward multidisciplinary and transdisciplinary data collection and decision making in all areas of disaster and crisis medicine and management [14].

Comprehensive risk management includes not only foreseeing its limitations and parallel achievement of readiness to react in the event of a threat, it also more reliably forecasts proper preparation of the society to take actions to protect it against potential threats [15,16,17].

## 3. Implementation

Currently, several national projects (or projects involving Poland) are being implemented in Poland in the field of natural disaster risk management. Again, these are the only crisis management projects that are currently being implemented and corresponding SFDRR Priorities are listed. None of the others listed have been fully implemented yet, therefore the listing of SFDRR Priorities are not feasible at this point in time, nor are available for assessment in this document: Those satisfactorily implemented to date are:

### 3.1. The Agricultural Drought Monitoring System

The project is led by the Institute of Soil Science and Plant Cultivation—State Research Institute on behalf of the Polish Ministry of Agriculture and Rural Development, and corresponds to SFDRR Priority 1. The Agricultural Drought Monitoring System (ADMS) is designed to identify areas at the level of municipalities, where potential losses occurred for crops due to drought conditions which are listed in the “Act on subsidies to insurance of agricultural crops and farm animals”. To designate the level of climatic water balance, statistic-empirical models of forecasts and simulation model for crop yields, developed at the Institute of Soil Science and Crop Cultivation—State Research Institute in Pulawy were used [18]. In determining the areas affected by the drought, besides the value of climatic water balance, the characteristics of the soil retention are determined by Soil category, and are identified based on soil and agricultural maps. In this way, a strong diversification of the susceptibility of Polish soil cover to the effects of a shortage of water is taken into account [18].

ADMS includes computer applications that are integrating meteorological data needed to calculate the climatic water balance along with data from soil-agricultural digital maps illustrating the spatial diversity of the various categories of water retention of agronomic soils. Information regarding the appearance of the drought in the form of sixty-day reports are forwarded to the Ministry of Agriculture and Rural Development and is then published on the website [18,19].

### 3.2. IT System of the Country’s Protection against Extreme Hazards

IT System of the Country’s Protection Against Extreme Hazards (Polish ISOK) aims to contribute to the resolution or minimization of several issues related to crisis resource management in Poland, with a particular focus on flood risks. These correspond to SFDRR Priorities 1 and 3. The main goal of the project is to be carried out through the inventory of available data resources, designing a system solution, building databases, risk and threat maps, and building and implementing an IT system, increasing public awareness of threats and crises. This goal is planned to be achieved by developing a flood hazard map, flood risk maps, maps of meteorological threats, maps of other hazards, and a map of Poland’s hydrographic distribution [20]. The project is mainly focused on the public. Anyone with a device which has access to the internet will be able to check if the area they are in is at risk of flooding, and if so, how big of a threat there is. In addition to individual recipients, project stakeholders are also to be responsible for spatial planning and flood protection planning. This mainly concerns the regional water management boards responsible for protecting areas at risk of flooding before they are populated in a way which hinders protection [20,21,22].

ISOK also means to address institutions responsible for preventing crisis situations. In particular, this applies to voivodeship crisis resource management centers and other government and self-government administration units, at the national, regional, and local level, dealing with issues of flood protection and other threats and crisis response. IT solutions are to significantly improve the flood and crisis management capabilities.

### 3.3. RescEU

This represents a European system aimed at combating natural disasters, which provides for the creation of a European-level force-reserve for civil protection, using tools such as airplanes, specialized water pumps, urban search and rescue facilities, field hospitals, and rescue teams. This corresponds to SFDRR Priority 4, although it only addresses preparedness for effective response and not the recovery phase. The force-reserves, designed to supplement domestic assets, are to be managed by the European Commission to support countries affected by disasters such as floods, forest fires, earthquakes, and epidemics. An operational example of the implementation of the system was the rescue operation of the Polish State Fire Service during forest fires in Sweden in 2018 [23,24]. The long-term aim is to add further capacities and assets and build up a stronger rescEU reserve. Europe has witnessed severe natural and man-made disasters in recent years: Forest fires, floods, storms, and earthquakes resulted in the loss of lives—more than 100 in 2018. To better protect citizens in need, rescEU reinforces the Union’s collective ability to prevent, prepare, and respond to disasters that affect our societies as of now [25].

The EU’s Civil Protection Mechanism is so far based on a system, through which the EU coordinates the voluntary contributions of Participating States to a country that has requested assistance. Offers of assistance are coordinated by the European Emergency Response Coordination Centre based in Brussels. In recent years, extreme weather conditions and new emerging threats have stretched the ability of Member States to help each other, especially when several Member States face the same type of disaster simultaneously. In such cases where there is limited or no availability of assets, the EU did not have a reserve capacity to assist overwhelmed Member States [25].

### 3.4. Urban Adaptation Plans

This project, which includes 44 Polish cities, is intended at adapting those cities to the observed and prognosed climate changes. Developed in cooperation with the Polish Ministry of the Environment, it covers approximately 30% of the population of Poland. These correspond to SFDRR Priorities 14. The project is designed to assess vulnerability to climate change and plan adaptation activities to counter the identified threats in cities with over 100,000 residents. This currently represents the only initiative of this type in Europe in which the ministry supports local authorities in coordinating activities adapting to the effects of climate change. Local urban plans for adapting to climate change (Polish MPA) will be created with the cooperation of authorities, disaster management experts, and residents, to find and operationally implement innovative adaptation solutions [26]. MPA implementation will change the everyday life of city dwellers. The modernized flood-protection system, effective water management procedures, or the development of the information and alarm systems in case of hazard will make the inhabitants feel safer. Aesthetic changes in the urban infrastructure and vegetated areas, reduced thermal hazard, improved living and investment conditions thanks to the urban space development plans shall improve the comfort of living in the cities, and reduce the risk resulting from the climate change effects.

The works on the plans will be carried out in line with the methodology developed by the consortium. The rhythm of works will be conditional on the rules of close cooperation between the urban and expert teams. An important component is also the participation of stakeholders and MPA beneficiaries at the same time who will be invited to participate in debates, e.g., during workshops and public consultations [26].

### 3.5. Government Centre for Security Alert

In case of impending danger, a population-based notification system will be utilized only when there is a high likelihood of life or health being threatened in a given area. These correspond to SFDRR Priorities 2 and 4. Government Centre for Security Alert (GCS Alert) is created on the basis of information on potential threats received from ministries, services (e.g., police, fire brigade, border guards), central offices, and institutions (e.g., Institute of Meteorology and Water Management—National Research Institute or voivodeship offices). A GCS Alert, a text notification from the Government Centre for Security (GCS), is one element of the early warning system. The information will mainly concern potential natural incidents linked to strong winds, intense rainfall, and violent storms. A GCS Alert will be activated only in the most serious of situations, as not all meteorological phenomena carry the risk of immediate threat. The alert is sent via a text message, generated by the operators of all networks based on information sent from the Government Security Center to persons in the area of potential danger, but limited to the specific poviat/county [22,27,28].

Encouragingly, in Poland, more and more is being discussed and debated about cooperation for effective climate risk management, and more insurers have taken on the risks associated with climate change, approaching it very comprehensively. At the same time, they have gathered more accurate data on how one can effectively manage risk. Numerous public institutions also run their activities in this area. One senses the legitimacy and progressive openness to the mutual exchange of data, information and knowledge, which should be interpreted as a testimony that a common focus on insuring the security of the state and its citizens, regardless of the circumstances, is emerging. This also includes civil planning implemented as part of the crisis management system, critical infrastructure protection standards, developing problems of business continuity management, as well as scientific and didactic activities [11,13,27].

## 4. Limitations of a Study

Current projects are in the implementation phase or have just been implemented, and it is difficult to consider the practical implementations, and even more so to evaluate the proper performance of the systems. The potential of these presently implemented systems will not stand still, but will need to adapt as sudden onset natural disasters increase in proportion to inherent climate changes. 

The authors have presented in other research the details containing the essential postulates, limitations concerning individual programs, and summaries of the current operational status of the implemented solutions [10,12,14,28]. The authors believe that this proposal is only the first step towards alleviating the consequences, which must also weigh in on the immediate debate between citizens and governments to ensure universal and equal opportunities to cover all traditional and newly exposed ‘vulnerable’ societies.

## 5. Conclusions

It must be emphasized that the Polish government remains oriented toward the emergency response phase of the disaster cycle (prevention, preparedness, response, recovery, rehabilitation) and has not properly embraced SFDRR goals fully. What remains, is a critically urgent and operationally important task to develop a risk management plan at the national level. This will not only contain a diagnosis in the area of disaster risk, but also determine the current administrative and organizational capacity, as well as available technical and financial resources. As part of the National Risk Management Plan, the selection of priorities in the area of risk management should be made together with the definition of key tasks and indicators to achieve the objectives. Current operational plans are not strategic and are quickly becoming obsolete. More emphasis should be put on predicting the negative effects of climate change and infrastructure changes [29].

Individual solutions, which have previously been agreed upon in a series of comprehensive studies will be highlighted in context of the project summary. There is a need to constantly build public awareness of the risk of natural disasters through formal education in schools and informally at the local level. Knowledge-based awareness helps to avoid areas at risk, to understand the importance of the warning received, to take decisions that are adequate to the situation, and react accordingly. It also allows you to make responsible decisions in the field of spatial planning, insurance, and reconstruction.

It is necessary to analyze whether individual initiatives affect the propensity to undertake preventive and adaptive actions at the level of local governments. For example, it is necessary to increase expenditures on information and educational activities that increase public awareness both in the area of threats related to the occurrence of natural disasters, as well as possible actions to reduce risk. Furthermore, there is a need to develop a systematic and homogeneous approach to the collection of statistical data, allowing to measure exposure, sensitivity and vulnerability to natural hazards in the social, economic and structural dimensions. This type of data (at least for the level of municipalities) can be collected and regularly shared by the Central Statistical Office as part of the existing Local Data Bank. The current available measurement infrastructure in Poland, as well as the limited density of its distribution, means that certain phenomena may not be captured.

It is recommended that State authorities be held accountable to publish cyclical and special (ad hoc) reports containing final estimates of losses caused by natural hazards, along with a precise geographical area. In the case of major disasters, it is necessary to specify in the report the exact course of events based on reports of services, victims and other entities (e.g., registrants of a given phenomenon). This must include, in addition, the applicable construction law, the so-called special acts in the area of infrastructure investments, and the effectiveness of construction supervision [30].

In Poland, as well as globally, critical importance is being placed on recognizing that these events and the appropriate response have as its core concept that they represent potential public health emergencies, which each and every component of government has an essential role. Only then will a mitigation of both direct and indirect mortality and morbidity and essential infrastructure loss be realized. Where applied globally, countries are already experiencing substantial risk reductions across many sectors of government and health [31]. Many developing countries would benefit from the incorporation of advances in science, technology and the development of a single framework that integrates disaster risk reduction, sustainable development, climate change adaptation and conflict prevention [16,29,32].
